# Wave types and energy conversion of impulse waves generated by landslides into mountain reservoirs

**DOI:** 10.1038/s41598-022-07993-9

**Published:** 2022-03-08

**Authors:** Linfeng Han, Pingyi Wang, Tao Yu

**Affiliations:** 1 School of Civil Engineering, Chongqing Jiaotong University, Chongqing, 400074 China; 2 National Engineering Research Center for Inland Waterway Regulation, Chongqing Jiaotong University, Chongqing, 400074 China

**Keywords:** Natural hazards, Ocean sciences, Energy science and technology, Engineering

## Abstract

Subaerial landslides sliding into shallow water are physically modeled in a three-dimensional wave basin. The generated impulse waves are highly nonlinear, and a large-scale splash zone is formed above the waves. Such impulse wave characteristics are different from those from landslides into deep water that are completely submerged after sliding. The recorded wave profiles included three wave types, namely nonlinear oscillatory wave, nonlinear transition wave and bore-like wave, mainly depending on the relative slide thickness and slide Froude number at impact. Bore-like waves were possible produced only by landslides into shallow water in three-dimensional experiments. The conversion rate of landslide kinetic energy at impact into the wave train energy is 1 to 18%. Energy conversion characteristics are compared with other two- and three-dimensional studies on landslide-generated waves and the results are discussed.

## Introduction

Landslides occurring at the bank of the reservoir can trigger impulsive waves that propagate both offshore and along the reservoir shoreline. The usually short propagation distance within mountain reservoirs leads to negligible wave attenuation thereby retaining the large damage potential for humans and the near reservoir infrastructure^1^. Damage caused by impulse wave run-up, for example, can extend to areas well-above the shoreline, endanger human life and cause major economic impacts. Dam overtopping can even result in reservoir failure and thus lead to catastrophic events. Historically, the highest wave run-up caused by impulse waves in reservoirs was observed in Vajont, North Italy in 1963. A rock flank failure of 250 × 10^6^ m^3^ volume slid into the water body, displacing almost the entire reservoir volume. The slide caused a wave run-up of about 200 m at the opposite shore^2^. A recent event occurred in 2008 in the Three Gorges Reservoir Region, China. An impulse wave of 31.8 min height generated by the Gongjiafang landslide with a volume of 380,000 m^3^ caused a maximum run-up of 12.4 m at the opposite shore^3^. Field data from historic events are limited to the landslide scarp, run-up trimline, far-field tide gauge recordings and the submarine deposit, where mapped. Hence, physical modelling is an important method for studying the wave generation, propagation and run-up.

Methods of simulating the landslide have included two-dimensional models with a solid block sliding down an incline^4–9^ or with granular materials^10–17^, and three-dimensional models using a solid block slide^18–25^ or a granular landslide^26–29^.

Although one order of magnitude smaller than tsunamis, the impulse waves in restricted waters (e.g., reservoirs, lakes and watercourses) may cause more serious consequences than ocean access. This is because in shallow water areas, the rock and soil mass has a stronger disturbance on the water body, and the behavior of the water is strongly nonlinear. In 2017 Huang et al.^30^, defined a partially submerged landslide after deposition as a landslide into shallow water. However, the previous research on landslide tsunamis mainly focuses on landslides into deep water that are completely submerged after sliding. Therefore, the present experiments were designed and conducted to fill this gap. Herein the attention is focused on the topic of wave type classification and energy conversion of landslide-generated impulse waves in shallow water in reservoirs.

## Physical model

### Experimental set-up

**Figure 1:**
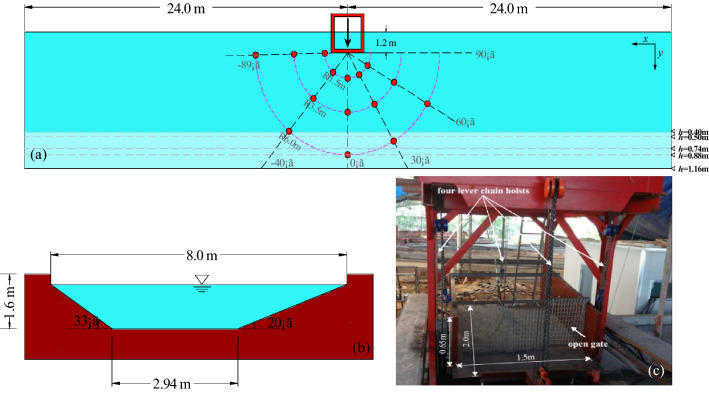
Sketch and picture of experimental set-up: (**a**) wave gauge array used to measure the water surface elevation of the landslide-generated tsunamis (red circles) with a water depth of *h* = 0.88 or 1.16 m. The gray dash lines correspond to water lines under different water depth conditions; (**b**) cross-sectional view of trapezoid dimensions for which water depth *h* = 1.6 m at bankfull flow; (**c**) chain hoist landslide tsunami generator.

The physical experiments on impulse waves generated by landslide into shallow water were conducted in a three-dimensional wave basin. The concrete wave basin is 48 m long, with a trapezoid cross-section, which has a depth of 1.6 m, a bottom width of 2.94 m, top width of 8.0 m, and both side slopes of 33° (left bank) and 20° (right bank), as shown in Fig. 1a,b. A chain hoist landslide tsunami generator was used to control the dynamic slide impact characteristics. The generator consists of a sliding box filled with up to 1.95 m^3^ of landslide material and four chain hoists that could adjust the precise location of collapse and hill slope angle effectively shown in Fig. 1c. As the gate opened, the landslide materials exited the slide box to accelerate solely by gravity towards the water surface. Landslide models with nine different volumes in the experiment were reported in Mu et al.^31^. The test program included a variation of the still water depth *h* = 0.40, 0.50, 0.74, 0.88 and 1.16 m, slide impact angle α = 20°, 40° and 60°. A total of 135 experimental trials were conducted that covered a wide range of source volumes and reservoir depths.

**Figure 2:**
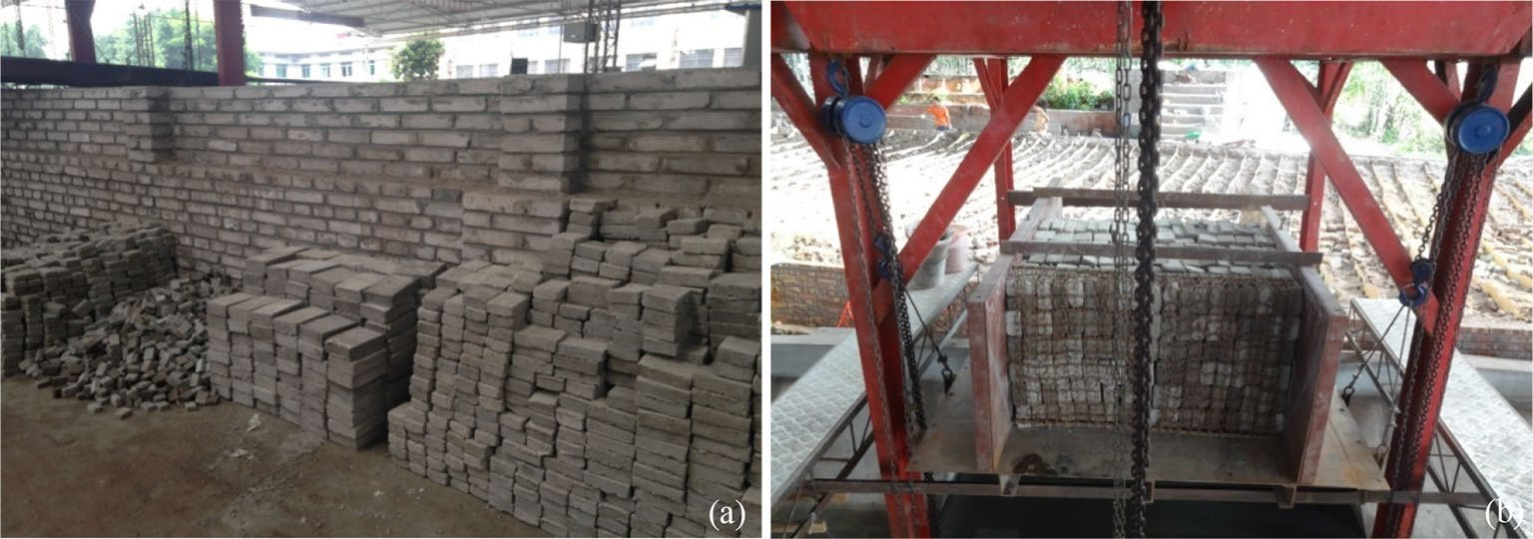
(**a**) Rigid concrete blocks; and (**b**) landslide model assembled with rigid blocks.

The naturally occurring landslide due to the degradation of rock mass in the water level fluctuation zone is one of the main forms of a reservoir-induced landslide in the later stage of water storage and one of the important paths of newborn landslide generation^32^. Because they may be large and very rapid, rockslides related to reservoirs generally have been considerably more destructive than slope movements in surficial materials. According to the crack developments of the rock mass in the Three Gorges Reservoir area, the rockslide generated waves were physically modeled with a combination of rigid blocks with various scales on a planar hill slope. The density of the consisted block was set to 2400 kg/m^3^ that matched the density of natural rock-soil masses, and the resulting rockslide model is shown in Fig. 2. Additional details on the experimental setup and rockslide evolution on the hill slope are reported in Han et al.^33^.

### Instrumentation set-up

A high-speed overhead camera and several above water side-view videos were deployed on the subaerial landslide motion region and impact location to capture the landslide kinetic characteristics at a frequency of 1000 frames per second (fps). A total of 15 ultrasonic wave gauges were installed in the wave basin to record the water surface profiles in radial and angular direction away from the landslide source. The gauge has a resolution of 1 mm and a recording frequency of 100 Hz. The wave probe located along the center line of wave basin at station *y* = 1.5 m was labeled as P1. In order to ensure the consistency of all tests, this paper sets the measured value from P1 gauge as the near-field maximum amplitude *a*_*m*_. The gauge locations in the wave basin at water depth of *h* = 0.88 and 1.16 m are shown in Fig. 1a. Since the recorded waves propagate over variable bathymetry, for low-water conditions, some gauges emplaced on the sloping walls of the wave basin cannot continue to work, so their position will be adjusted appropriately.

## Observations and analysis

### Impulse wave generation

The wave characteristics are dependent on the landslide impact parameters. The governing parameters for impulse waves generated by rock landslides in this study are the slide impact velocity *v*_*s*_, still-water depth *h*, slide thickness *s*, slide width *w*, slide volume *V*_*s*_. The corresponding dimensionless parameters are the slide Froude number *F* = *v*_*s*_/(*gh*)^0.5^, relative slide width *W* = *w*/*h*, relative slide thickness *S* = *s*/*h*, relative slide volume *V* = *V*_*s*_/*h*^3^. When the gate opens rapidly, the landslide material is released from the slide box and declines along the hill slope under gravity while decreasing the slide thickness and increasing the slide length and width. The porosity will increase due to the separation of rock mass during sliding. Across the bulk of the landslide width, the velocity is mostly uniform. The landslide front velocity is measured with side-view video and high-speed overhead camera. The velocity at impact is in the range 0.823 < *v*_*s*_/(*gs*_0_)^0.5^ < 1.836 for the landslide volumes of *V*_*s*_ = 0.1 m^3^ to 0.9 m^3^, where *s*_0_ is the initial slide thickness and *g* is the gravitational acceleration.

**Figure 3:**
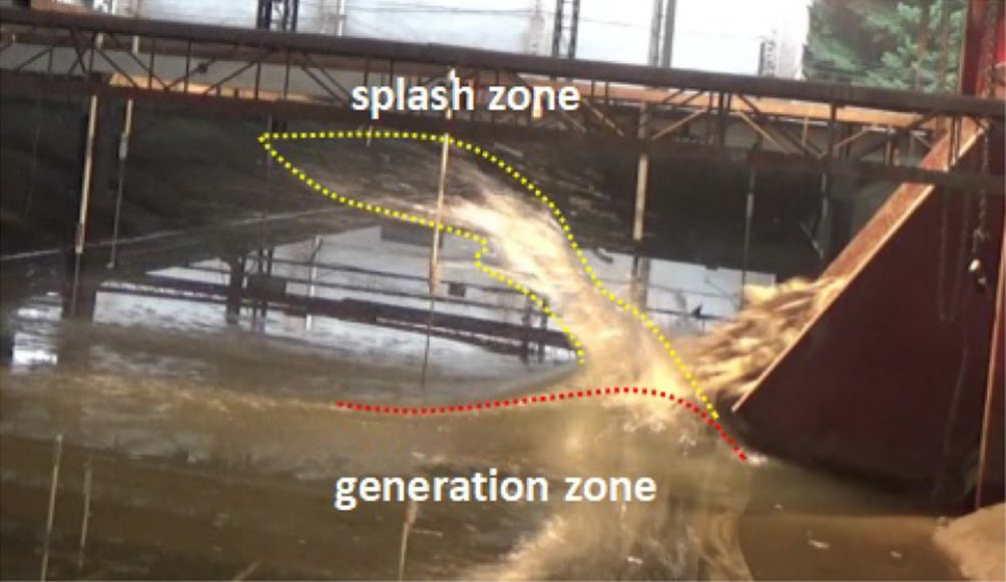
Photographs of propagating leading wave crest and water splashing in the near-field zone.

Landslide-generated impulse wave is a complex phenomenon caused by the multi-phase interaction of slide mass, air and water^34^. In this experiment, the generation area of the near-field wave is within the range 0 ~ 1.45 m from the impact point. Immediately after the impact begins, the water is pushed up vertically by the slide and accompanied by splashing with the result that a surface splash zone is formed above the wave generation zone, as shown in Fig. 3. In shallow waters, landslide deposits lead to dramatic changes in channel topography, and the water body is quickly squeezed out of the water surface to form a water-jet. If the distance between the two sides of the reservoir or channel is short, the water-jet is likely to directly hit the opposing hillslope. As a dissipative item, the water body in the splash zone does not contribute to the wave generation, resulting in a low energy conversion rate from landslide to impulse waves in shallow-water areas, and a large part of the kinetic slide energy is consumed in the splash zone.

### Wave energy conversion

The energy conversion describes the kinetic slide energy upon impact transferring to the generated wave energy. The kinetic slide impact energy is given as $E_{s} = (1/2)m_{s} v_{s}^{2}$, where $m_{s}$ is the slide mass,$v_{s}$ is the slide velocity at impact. The impulse wave energy involves two forms: kinetic energy $E_{kin}$ and potential energy $E_{pot}$. The wave potential energy per unit width of the recorded wavefront profiles is given as

1 $$ dE_{pot} = \frac{1}{2}\rho_{w} gc\int_{0}^{T} {\eta^{2} dt} , $$

where $\rho_{w}$ is the water density, *c* is the wave celerity, $\eta$ is the water surface displacement. In the three-dimensional model, the impulse wave propagates in the form of a radial wave front, so the total wave potential energy of a radial wavefront at propagation distance *r* in three-dimensional cylindrical coordinates ($- \pi {/2} \le \theta \le \pi {/2}$) can be expressed as

2 $$ E_{pot} = \int_{ - \pi /2}^{\pi /2} {\left( {\frac{1}{2}\rho_{w} gc\int_{0}^{T} {\eta^{2} dt} } \right)} rd\theta . $$

**Figure 4:**
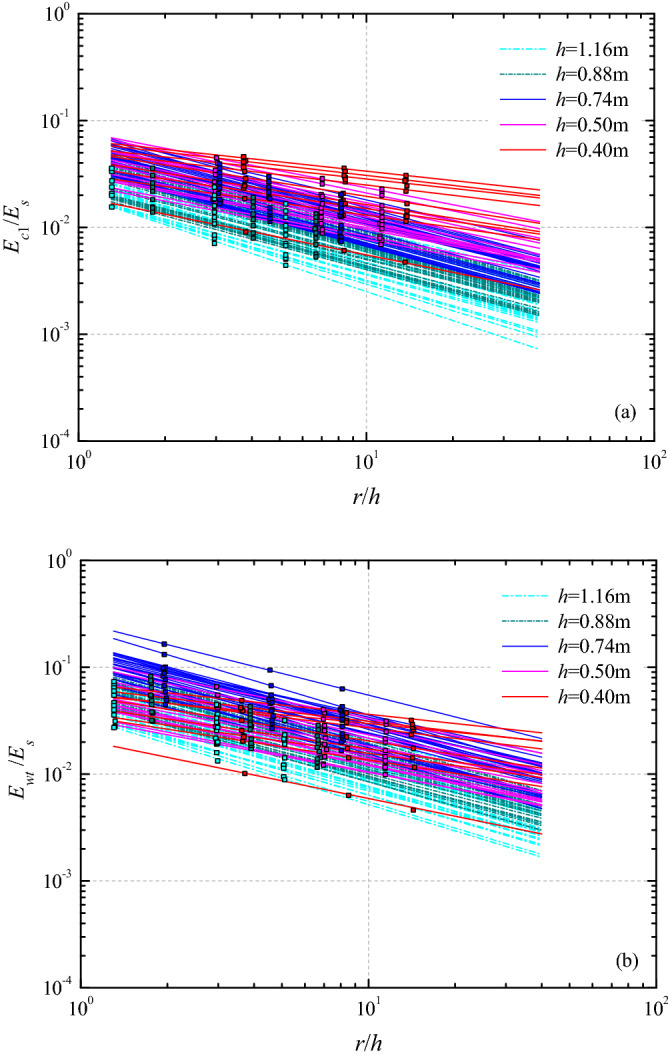
Attenuation of wave energy relative to kinetic slide impact energy with varying water depth for the (**a**) leading wave crest *E*_*cr*1_/*E*_*s*_ and (**b**) wave train *E*_*wt*_/*E*_*s*_.

In the case of only considering the initial wave crest potential energy, the integral range starts from zero and ends at the first down crossing point. Since the amplitudes of the trailing wave train are much smaller than that of the leading wave, the energy packet contained in the first three waves is used to computing the wave train energy in this paper. Mohammed and Fritz^26^ obtained the wave potential interpolation function of the leading wave crest by three-dimensional subaerial granular landslides, as follows

3 $$ dE_{pot} \left( {r,\theta } \right) = k_{{E_{c1} }} r^{n} \cos^{2} \theta $$

within the wave propagation range $0 \le r \le r_{\max }$ and $- \pi /2 \le \theta \le \pi /2$, where *n* represents the amplitude decay rate. Since the movement of water particles in the water column is difficult to measure, the generated wave kinetic energy cannot be directly estimated. At present, the equipartition assumption ($E_{kin} \approx E_{pot}$) in linear waves is often used to calculate total wave energy. In 1985 Williams^35^, found that when the solitary wave height is close to the breaking limit, the total wave energy ($E_{tot} = E_{pot} + E_{kin}$) obtained by numerical calculation may exceed 11% of the equipartition assumption. But this result is typically only a few percent in the current study. Therefore, the total wave energy of the leading wave crest can be expressed as

4 $$ E_{cr1} = \int_{ - \pi /2}^{\pi /2} {\left( {\rho_{w} gc\int_{0}^{{T_{cr1} }} {\eta^{2} dt} } \right)rd\theta } , $$

where $T_{cr1}$ is the period of the leading wave crest from the initial rise to the first down-crossing. As shown in Fig. 4a, the energy conversion from kinetic slide impact energy to leading wave crest with a rockslide on a planar hill slope is between 0.5 and 7%. Under the influence of wave energy dispersion and frictional dissipation, the energy of the initial wave crest decreases with the propagation distance. Since the wave celerity of the waves in the wave train is different, the total wave train energy can only be obtained by the superposition of the wave energy of the each individual wave amplitude. For landslide-generated waves, the wave energy of the trailing wave train is negligible relative to main wave. In the present experiments, the energy packet contained in the first three waves is used to calculate the wave train energy. The energy conversion from kinetic slide impact energy to wave train with a rockslide on a planar hill slope is between 1 and 18% as shown in Fig. 4b. Table 1 includes studies in which the energy conversion between landslide kinetic energy and generated wave energy from different model types were investigated. From Table 1 the energy conversion from kinetic energy at slide impact to wave energy in the 2D models is much greater than in the 3D models. The 2D models result in high energy conversion rates as lateral constraint on the landslide motion and wave generation. In contrast, the 3D models don’t have lateral constraint on landslide and water body, which increases granular landslide deformation and triggers lateral escape of water body, thereby decreasing the energy conversion from landslide impact to generated waves. In addition, the energy conversion rates are slightly larger in this study than in the 3D experiments of Mohammed and Fritz^26^ and Mcfall and Fritz^29^. Compared with 3D rock slide models, the 3D granular landslides undergo greater slide deformation during the subaerial and subaqueous movement, thereby dissipating more slide energy through internal frictional effects and decreasing the energy conversion during impact.

**Table 1:** Experimental model studies of energy conversion between the landslide kinetic energy and the generated impulse waves.

Study	Model type	*E*_*c*1_/*E*_*s*_	*E*_*wt*_/*E*_*s*_
Kamphuis and Bowering^4^	2D block model	–	10–50%
Huber^36^	2D granular slide model	–	1–40%
Watts^6^	2D block model (underwater)	–	2–13%
Fritz et al.^12^	2D granular slide model	2–30%	4–50%
Ataie-Ashtiani and Nik-Khah^37^	2D block model	–	5–50%
Heller and Hager^15^	2D granular slide model	–	11.3–85.7%
Mohammed and Fritz^26^	3D gravel slide model	0.5–3%	1–15%
McFall and Fritz^29^	3D cobble slide model	0.5–11%	1–24%
Current study	3D rock slide model	0.5–7%	1–18%

In the energy conversion process of sliding energy to wave train, the energy conversion rate in shallow water area is generally lower than that in deep water area. This is because it takes a short time form landslide impact water body to initial wave generation. Therefore, when the slide volume is relatively larger than the receiving water body, the landslide will deposit rapidly after entering the water, resulting in a large amount of slide impact energy that cannot be transformed into impulse waves. In addition, due to the incompressibility of the water body, the part of the energy that is not involved in wave making forms a water jet-flow with a lot of splashing above the wave generation zone, as shown in Fig. 3. As a dissipation term, the water body in the splash zone does not participate in the wave making process, which leads to a lower energy conversion rate in shallow-water areas than in deep-water areas.

### Wave profiles

According to the study’s results of two-dimensional block slide experiments by Noda^38^ and two-dimensional granular slide experiments by Fritz et al.^12^, impulse waves generated by landslides were classified into nonlinear oscillatory waves, nonlinear transition waves, solitary-like waves and dissipative transient bores based on the landslide Froude number and relative slide thickness at impact. However, Mohammed and Fritz^26^ and MacFall and Fritz^29^ only found two wave types, nonlinear oscillatory and nonlinear transition by their three-dimensional experiments. Solitary and bore-like waves were not observed in above studies because the additional degree of freedom in three-dimensional granular models increases landslide deformation, thereby reducing the slide thickness at impact.

**Figure 5:**
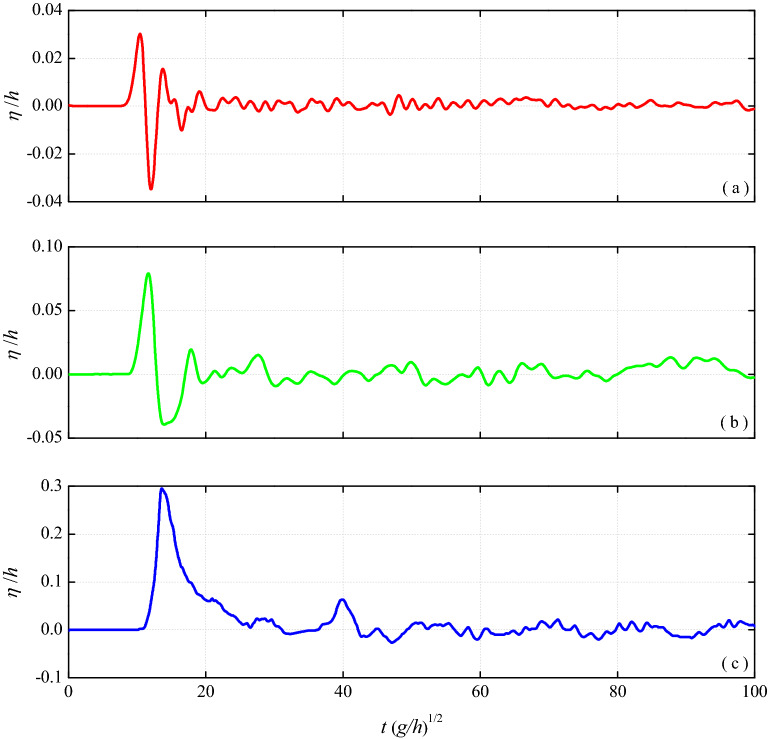
Illustration of three types of waves observed by P1 probe: (**a**) nonlinear oscillatory wave profiles with slide parameters *F* = 0.74, *S* = 0.17, *V* = 0.19, *h* = 1.16 m; (**b**) nonlinear transition wave profiles with slide parameters *F* = 1.63, *S* = 0.81, *V* = 2.22, *h* = 0.74 m; (**c**) bore-like wave profiles with slide parameters *F* = 2.13, *S* = 1.5, *V* = 9.38, *h* = 0.4 m.

In the present three-dimensional experiments, we have not only observed nonlinear oscillatory and nonlinear transition type of waves but also observed bore-like waves in some cases of landslides into shallow water. Figure 5a shows a recorded nonlinear oscillatory wave profile with slide parameters *F* = 0.74, *S* = 0.17, *V* = 0.19, *h* = 1.16 m. This wave type has a leading main wave crest followed by a strong dispersive oscillatory wave train, and the strong dispersion characteristic can stretch the wave train and transiently enhance trailing waves during propagation. This study found that nonlinear oscillatory waves are generated by relatively slower and thinner landslides. Figure 5b shows a recorded nonlinear transition wave profile with slide parameters *F* = 1.63, *S* = 0.81, *V* = 2.22, *h* = 0.74 m. This wave type is characterized by a main leading wave crest and a long shallow trough followed by a weakly dispersive wave train. The experiment found that nonlinear transition waves are generated by relatively faster and thicker landslides. Figure 5c shows a recorded bore-like wave profile with slide parameters *F* = 2.13, *S* = 1.5, *V* = 9.38, *h* = 0.4 m. Compared with other types of waves, the energy conversion from impacting slide mass to generated bore-like wave is energetically inefficient. Nevertheless, the bore-like waves generated by landslides often have a relatively large amplitude in the near-field area because these waves are characterized by steep wave front, flat wave tail and entraining amount of air. Heller and Hager^15^ found that the near-field maximum amplitude of bore wave can be as high as 2.5 times the still water depth *h*. The observed bore-like waves were in intermediate to shallow water range and generated by fast and thick landslides. Therefore, for impulse wave events generated by landslides into shallow water such as reservoirs and lakes, more attention should be paid to the possible bore-like waves.

**Figure 6:**
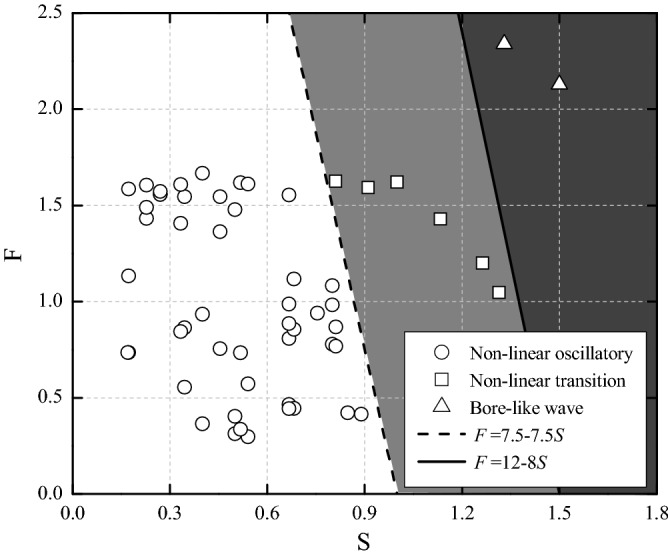
Observed wave type classification based on the slide Froude number *F* = *v*_*s*_/(*gh*)^0.5^ and relative slide thickness *S* = *s*/*h*.

All observed wave trains expand outward as the propagation distance due to nonlinearity and dispersion. The leading waves attenuate as the propagation distance, while dispersion temporarily enhances subsequent trailing waves^39^. In this study, the observed three wave-type regions are shown in Fig. 6. The wave-type region was determined by the slide Froude number *F* and the relative slide thickness *S*. Compared with three-dimensional granular landslide experiments by Mohammed and Fritz^26^, the transition from nonlinear oscillatory to nonlinear transition wave-type region in present study requires relatively higher values of dimensionless parameters *F* and *S* (*F* = 7.5–7.5*S*). Bore-like waves were generated by landslides with larger values of *F* and *S* than those producing a nonlinear transition wave. A bore-like wave as the leading wave was observed if the slide Froude number satisfied the empirical relationship *F* ≥ (12–8*S*). Hence, the bore-like waves were generated by thick landslide relative to water depth at a large slide Froude number.

## Conclusions

Based on the Froude similitude, impulse waves induced by three-dimensional rock landslides are physically modeled, and the wave types and energy conversion of subaerial landslide generated impulse waves were investigated. The main results may be summarized as follows:

1. Compared with two-dimensional models, three-dimensional granular landslides have a larger amount of deformation due to the lack of lateral constraints during slide, thus reducing the effectiveness of energy conversion and wave generation. Between 0.5 and 7% of the landslide kinetic energy is converted to the leading wave crest and 1 to 18% is converted the wave train. For the cases of landslides into shallow water, big wave splash forming above the wave generation zone consumes a large amount of landslide energy, resulting in a smaller energy conversion than a landslide into deep water.
2. Three wave types were observed: nonlinear oscillatory wave, nonlinear transition wave and bore-like wave, depending mainly on the relative slide thickness *S* = *s*/*h* and the slide Froude number *F* = *v*_*s*_/(*gh*)^1/2^. Nonlinear oscillatory waves result generally from small dimensionless parameters *F* and *S*. They consist of a leading main wave crest followed by a strong dispersive oscillatory wave train. Nonlinear transition waves involve generally medium to large dimensionless parameters *F* and *S*. They consist of a major leading wave crest and a long shallow trough, followed by a weakly diffuse wave train. Bore-like waves result generally from large dimensionless parameters *F* and *S.* They consist of one dominant wave with a large amount of air at the wave front. In three-dimensional experiments, only landslides into shallow water could produce bore-type wave profiles.

Impulse wave events generated by landslides into shallow water occur widely in restricted waters such as reservoirs, lakes and watercourses, and such impulse waves often cause large disasters. The classification of impulse waves in shallow water may be simply applied to practical predictions since it depends directly on basic parameters, which is useful for disaster mitigation of reservoirs.
